# Perspectives of pharmacists in general practice from qualitative focus groups with patients during a pilot study

**DOI:** 10.3399/BJGPO.2021.0112

**Published:** 2022-03-09

**Authors:** Claire Mann, Claire Anderson, Matthew Boyd, Yasmin Karsan, Tristan Emerson

**Affiliations:** 1 Centre for Health Innovation, Leadership and Learning, Nottingham University Business School, University of Nottingham, Nottingham, UK; 2 School of Pharmacy, University of Nottingham, Nottingham, UK; 3 School of English, University of Nottingham, Nottingham, UK

**Keywords:** general practice, pharmacists, patients, medicines optimisation, medication review, primary healthcare

## Abstract

**Background:**

Utilising skill mix in general practice is proposed as a solution to the demand–supply issue. Pharmacists can play an important role in this context, leading to an increase in training and funding for independent prescriber roles. A role for pharmacists in general practice was funded, piloted, and evaluated by NHS England (NHSE) from 2015.

**Aim:**

To answer the following question: what is the patient perspective of pharmacists in patient-facing roles in general practice in the UK?

**Design & setting:**

Focus group interviews exploring patient perspectives on the pharmacist role in the UK.

**Method:**

Thirty-four patients were interviewed in five focus group (January–December 2016). Data were iteratively analysed using the one sheet of paper technique.

**Results:**

While the public were aware of the primary care crisis, they were less well informed about potential solutions. Data showed patients primarily sought access to a clinician over expressing a preference for any type of clinician. Low awareness was shown about the pharmacist role and there was initial confusion about it. Acceptability levels were high. It was found that pharmacists added value and provided an expert medication-focused service, which had a positive impact on medicines use. Patients reported benefit from longer appointments, feeling they were not rushed, and that all their conditions were being considered holistically. They trusted pharmacists as experts in medication and trust was consolidated over time. Regular coaching from a pharmacist could lead to improved patient self-monitoring and self-care.

**Conclusion:**

Pharmacists can add value to the general practice team and this is recognised by patients.

## How this fits in

Due to the relative novelty of patient-facing role for pharmacists in general practice, there is limited research published in this field on the patient perspective. This article contributes to understanding of the role from the patient's point of view, which — to the authors’ knowledge — has only been reported in one other study. This research helps inform those working in general practice about patient perspectives on this role.

## Introduction

Pressures on primary care in the UK are well documented.^1,2^ Many of the consultations in general practice (family practice), which average at six per year per person, are routine issues concerning medication.^3^ Utilising a wide skill mix in general practice has long been proposed as a solution to the demand–supply issue.^4–6^ Pharmacists can play an important role in this context^7,8^ leading to an increase in training and funding for independent prescriber roles.^9^ A role for pharmacists in patient-facing roles in general practice was funded, piloted, and evaluated by NHSE from 2015.^10^

The NHSE-commissioned evaluation report^10^ examined the role and its impact, and the authors' realist review^11^ identified some key questions to be addressed. The effectiveness of the role has also been evaluated.^12–14^ There are multiple insights into perspectives on the role from pharmacists,^15–17^ GPs,^18^ and the multidisciplinary team.^15,17^ However, there is limited data reported from the patient perspective. Early patient views suggested a broad acceptability of the role with some initial resistance.^19^ However, the lack of peer review, limited sampling approach, and a low participation and completion rate limited generalisability. One exploratory study of 20 interviews with patients in one area has given some initial insights into the views of a small group of patients’.^20^ The present work builds on this by exploring a wider range of patient views in response to the role. The aim was to answer the research question: what is the patient perspective of pharmacists in patient-facing general practice in the UK? This research work was undertaken in the pilot phase of the scheme in 2016 and may provide a useful benchmark as the role progresses.

## Method

Focus group interviews explored patient perspectives on the pharmacist role. Thirty-four patients participated in five focus group interviews with two researchers (CM, LB) in January–December 2016.

The inclusion criteria for participants defined them as patients at a general practice where a new pharmacist role was being trialled. Purposive and opportunistic sampling approaches identified key participants. Practice managers acted as gatekeepers organising the focus groups. All patients were given the same information and were aware that they were contributing to the evaluation of the new role.

The project researcher (CM) conducted the focus groups, each lasting approximately 1 hour. The researcher was well known to the practice staff teams as she conducted an ethnographic research at each site including participant observations.

**Figure 1.**
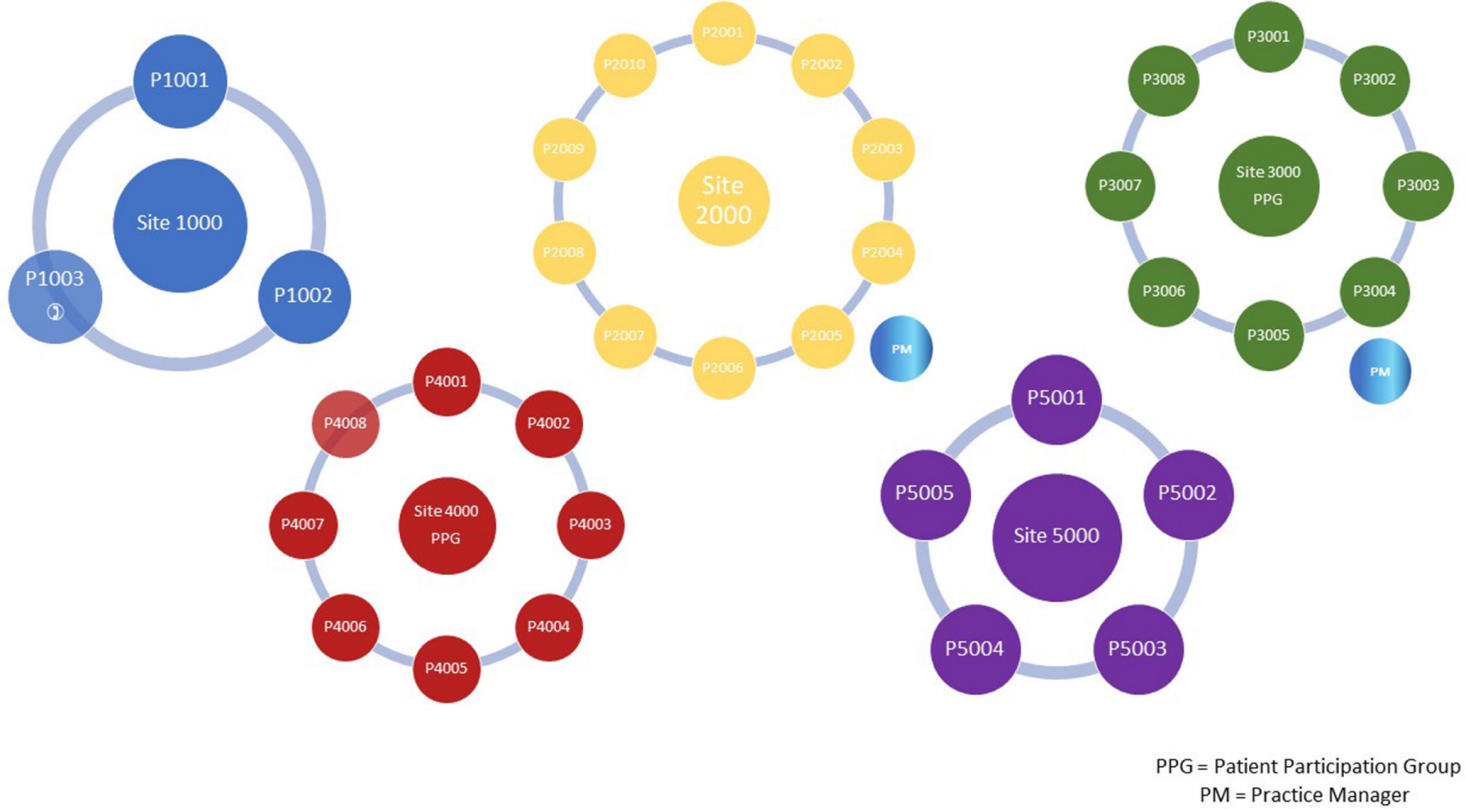
Distribution of patient focus groups. PPG = patient participation group. PM = practice manager.

Figure 1 summarises the participants in each focus group.

The demographics of the patients are outlined in Table 1.

**Table 1.** Demographics of patient participants

Sex	
Male	15
Female	18
Total	**33**
**Age** **, year** **s**	
>60	22
40–60	9
<40	2
Total	**33**
**Ethnic** **group**	
White	29
BAME	4
Total	**33**

Missing data *n* = 1. BAME = Black, Asian, and Minority Ethnic.

A broad topic guide was developed by two researchers (CM, MB). A range of common questions and the semi-structured nature of the interviews ensured that all participants had the opportunity to fully reflect on their experience of the pharmacist role and contribute to the evaluation.

Interviews were digitally recorded and transcribed verbatim (by LB); then anonymised, and thematically analysed by one researcher (CM) using NVivo (version 12) software. The data were primarily collected as part of a wider ethnographic service evaluation. Key themes were identified by four researchers (CM, CA, YK, TE) using Ziebland and McPherson's^21^ one sheet of paper technique. The team conducted independent analysis followed by an discursive analysis approach, which allowed for iterative development of themes improving the rigour of the work through intercoder reliability.^22^

## Results

The following sections highlight the key themes emerging from the patient voice. In these results the term ‘pharmacist’ is used to refer to pharmacists who are independent prescribers working in general practice, as opposed to community pharmacists.

### Access, awareness, and acceptability

Patients showed an awareness and understanding of the primary care crisis and pressures on GP time. Patients at every site recognised the release of GP capacity as a clear benefit of the scheme:

*‘* *You know, the National Health Service is in trouble, they say they want the doctors to do more, the doctor can’t do more than* *8* *–* *9* *hours a day* *. I don’t know how many patients they are seeing now but there is a fine line to what they can do. So, anybody who can give them the help … it has got to be better for everybody.* *’* (P2007)

*‘It was extremely helpful but more importantly, the times that you are able to see CP1 to discuss medication, you are not taking up a GP’s appointment.* *’* (P3001)

The majority of participants (*n* = 31) had experienced a consultation with a pharmacist at their GP practice. Most patients were aware that they would see a pharmacist and had no objections:

*‘The receptionist told me if I was alright seeing the pharmacist, and I said yes.’* (P2008)

Two patients reported that when they had been informed that their appointment was with a pharmacist, they had assumed this meant the pharmacist at the community pharmacy located in the practice:

*‘Well to be honest when I accepted the appointment, part of me thought I was just going to go to the chemist at the bottom and just speak over the counter.* *’* (P5002)

Several patients contacted their surgery for an appointment and were invited to see a pharmacist. Acceptability levels were high, and no negative reactions were reported:

*‘So, I rang up to make an appointment and she said do you want to see the pharmacist or the doctor? I said well everybody is as good as everybody else otherwise they wouldn’t be in a job, would they?* *’* (P5003)

Acceptability of the role was high among all patients and transferred into positive messages among the local community:

‘*… it very quickly became known within the local community that there was a pharmacist there. Because people felt they were going away with something good. I live in the community, I have worked in this community for 30 years so I know a lot of people, and a lot of people were saying oh the pharmacist is good … And I went oh good, what is happening, what are you liking about it? Oh, you get a 20-minute appointment up at the doctors! People were saying they understand the medication more, they know why they are taking it. Some of them had come away saying I don’t have to take such and such anymore. I am not saying every single person has been elated by it but my first introduction of it was it is really good and that is from outside on the street.’* (P4002)

*‘* *I have mentioned it to quite a few of my friends up and down the country, they haven’t got one, they say* *"* *oh that’s a good idea isn’t it?* *"* *So other people can see the benefits of it* *.'* (P2005)

The data above show that the public are aware of the primary care crisis, but they are less aware of potential solutions such as the pharmacist role. The data showed patients primarily sought access to a clinician over expressing a preference for any type of clinician. Low levels of awareness were shown about the role, and there was initial confusion between the pharmacist in the practice and the community pharmacist role. Acceptability levels were high and the ‘word on the street’ was positive.

### What pharmacists in general practice do

A high proportion of patients suggested that their initial consultation with the pharmacist was either a medication review required to issue a repeat prescription or a check-up for a long-term condition, such as a blood pressure check. For many patients the initial appointment combined these and several also included additional medication queries. Data suggested that pharmacists were able to conduct thorough discussions of medications use and medicines optimisation tasks. A benefit of the pharmacist being an independent prescriber was that they could alter prescriptions for patients. There was evidence that the outcome for patients was increased understanding of their medication:

*‘She went through all my medicines, actually explained what they were for, why I should take them. I thought it was a good idea. Normally all you do is get into your review, say yes and it is done. With* [pharmacist CP3] *you can say well why am I taking them, what for?* *’* (P3002)

The positive outcomes of the appointments with pharmacists were varied and wide ranging. Several patients reported improved use of medication including deprescribing, improvements to medication cycles, and improvement in adherence, and as a result a range of benefits related to physical wellbeing and quality of life. P1001 had been given the wrong directions for her overactive bladder medication and as a result was waking every hour in the night. Adjusting her medication dosage resulted in a much-improved sleep pattern. P2005 was given advice on correct use of his inhaler, which got rid of a persistent cough. P2009 had her eye medication changed and this got rid of symptoms of red, itchy, dry eyes, which had led to her being unable to wear make-up for a period of years.

Patients suggested that when they had increased understanding this would lead to increased adherence:

*‘His advice to me, like I say I tend to bit and bob a bit with my tablets, wait for the pain to come and then take them, when I should be taking them regularly every day. I think he had got a diagram up on the screen to show me* *… He showed me a diagram and said this is what will happen if you just take them as and when, it will ease it for a little bit, but if you take them regular then it stays in your body and it is there all the time.* *’* (P5002)

Data showed that despite having a medication focus, pharmacists also added value through holistic skills and promoting healthy lifestyle advice and positive change:

*‘Yes, I cut salt out, I’ve nearly cut chips out … I’ve cut pastry out, he said do it, so you have got to do it haven’t you?* *H* *e said all that is adding up to your blood pressure, it’s adding up to your cholesterol, cut all that out and hopefully get it down*.’ (P5003)

*‘So, he said I had got to make sure I had plenty of water, a litre of water a day. The best way of making sure that you have it is getting a bottle, just fill it up, and drink it through the day.* *H* *e kind of explained that the counter effect it can have a bad effect on your kidneys, I didn’t really appreciate that before.* *’* (P5005)

### How they do it

Early consultations and annual medication reviews can require longer appointments, which patients appreciate and benefit from:

*‘You were able to get some quality time with a clinician. Because obviously with the GPs you get* *10* *minutes* *and you try and squeeze everything into that. With* [pharmacist CP4] *you got about* *20* *minutes* *, or sometimes over* *20* *minutes* *and it was not just with medication, it was to do with things like lifestyle issues, with the best will in the world, GPs can’t tackle in* *10* *minutes* *.* *’* (P4001)

There is, however, also evidence that pharmacists have a unique approach and consultation style that enables them to undertake clinical work and also add value. Several patients commented on their satisfaction with the style of the consultation:

*‘I didn’t feel any rush at all. I could talk about everything and he could explain things. He took his time explaining absolutely everything.’* (P1002)

*‘I felt that he had a little bit more time than you generally find with the doctor. He was able to discuss how are you getting on with the tablets, are you taking them on a regular basis.* *’* (P5002)

There was evidence that patients felt that the pharmacist offered a holistic service seeing them as a ‘whole person’:

*‘It was actually a really long consultation, really thorough. I think probably looked at my mum as a whole, even though she wasn’t there. I actually felt that he knew my mum even though he didn’t. When I say didn’t, he understood her medication and he even went through one of her blood tests with me. Because she was taking ibuprofen, he actually said really she would be better with a gel because of it affecting her kidney function*.’ (P1002)

*‘I mean with my medication before, it was just a case of the doctor thinking oh yes, she needs so and so, adding it to the prescription. I never felt that they had time to look at the whole lot.* *’* (P3001)

Patients reported that they trusted the pharmacist, especially as experts in relation to medication:

‘*She knew all the medication I was on, and how one would react with another. So, she was able to make sure that everything that I was having was correct for me.’*

Pharmacists used motivational interviewing techniques that encouraged patients. Patients built trust through developing relationships and regular appointments with the pharmacist. Several patients at one site reported being educated about their blood pressure, and moved towards more self-care and less reliance on primary care by being encouraged to report regular home readings to the pharmacist.

While longer appointments benefit patients, the strength of pharmacist consultations is in their consultation style, which made patients feel unhurried, and all their conditions were being considered holistically. Patients suggested trust was solidified through regular appointments and relationship building, and there was evidence that regular coaching from a pharmacist could lead to improved patient self-monitoring and self-care.

### The pharmacist in the GP multidisciplinary team

Patients reflected on the role of the pharmacist within the broader multidisciplinary team. Patients appreciated the benefits of teamwork and selecting the right clinician for the right task:

*‘So* [pharmacist CP1] *has been working with* [nurse 1] *and* [healthcare assistant 1] *first of all, in like a threesome, to get my tachycardia so that it wouldn’t be a problem. So, I have been seeing her* [pharmacist CP1] *regularly. I find it is a combination of everybody really because I can’t remember the last time I saw the doctor. It was either* [pharmacist CP1]*, or* [nurse 1]*. Between the three of you, you have all sorted me. It’s very rare I bother the doctor. You are doing him out of a job.* *’* (P2005)

Some patients gave examples of the way the pharmacist added value to the team by helping them in ways that others had not been able to. One patient had seen both a nurse and a doctor with a rash, but it was only resolved by the pharmacist as they identified it was an adverse reaction to a medicine and worked with the patient to trial alternatives.

### Pharmacists in the community pharmacy

Initially there was some confusion between the patient being told they would see a pharmacist and understanding that it would be in the practice rather than in the community pharmacy. One person (P205) pointed out the pharmacist they saw was the same person they had seen as their community pharmacist previously. Several patients reported having a positive relationship with the pharmacist:

*‘I was quite happy with that because over the years, I have gone to a pharmacist first if I had got a problem. Quite often they have been able to do something without having to see a doctor anyway. So, when there was a pharmacist here, I thought that’s fantastic.* *’* (P2010)

Some patients suggested they would be happy to have the same service in a community pharmacy:

*‘I would if, my view is if you have got somebody with an equivalent standard or that sort of standing, they have got the necessary room for confidentiality that they could see you, then I have no problems with it.* *’* (P4005)

By contrast, some patients also suggested that they would not be comfortable having the service delivered outside of the practice. For some, this related to access to electronic patient records:

*‘* *It’s all in one house isn’t it, your records are all in the one place. If you went to a pharmacist and they altered your medication at Boots, it is not* *100* *%* *sure he is going to let the doctors know here. So, you come in for a repeat prescription and you are not getting the same.* *’* (P2007)

Some patients suggested that the practice was a comforting environment where they had the opportunity to build relationships. Others suggested they felt safe in the general practice space owing to the co-location of doctors and nurses.

## Discussion

### Summary

The study contributes to early insights into the patient perspective on the role of the pharmacist in general practice. Patients primarily sought access to a clinician (over expressing a preference for any type of clinician). There was low awareness and initial confusion about the pharmacist role but acceptability levels were high. Patients reported benefit from longer appointments, feeling they were not rushed and that all their conditions were being considered holistically. Pharmacists provided an expert medication-focused service, which had a positive impact on medicines use. Patients trusted pharmacists as experts in medication and trust was consolidated over time. Regular coaching from a pharmacist could lead to improved patient self-monitoring and self-care.

### Strengths and limitations

The strength of this study is the patient voice on a new role. This work was undertaken as a component of the NHSE evaluation of the piloting of roles for pharmacists in general practice and findings of the broad study were used to inform further commissioning and development of this role.^10^

The main limitation of the study is that it was undertaken during the pilot phase of the role and significant time has passed, over which the role has changed and developed. While the results are valid, it is likely perspectives may have changed as the role is now embedded. Furthermore, the recent pandemic will also have impacted patient views on both general practice and pharmacist services. It will be important to follow-up with further studies on the patient perspective to investigate whether more recent views represent any change in the perspectives presented here.

A limitation of the study may be that most of the patients were aged >60 years and from a White ethnic group, and further studies should ensure a wide demographic of participants.

There were a number of limitations in using the focus group methodology; for example, one group was small ( three participants), which would not usually be classified as ‘group’ discussion. However, the interview was an interactive discussion between patients and yielded more insight than individual interviews, and so was accepted as valid group interview data. At two sites, practice managers insisted on attending the group discussion, and this could have impacted the power dynamics and type of data that the patients chose to share.

### Comparison with existing literature

The results of present study show that patients are happy getting access to the right person at the right time. This is the key aim of NHS Improvement and the NHS RightCare programme.^23^ This finding is consistent with other studies such as Marques *et al*'s (2018)^24^ review of a similar patient-facing role for pharmacists, and also consistent the views of other stakeholders such as GPs and pharmacists in other studies,^15,17,20^ The findings are also consistent with findings from other studies suggesting that other roles in general practice can make unique contributions to the team, including nurses,^25–27^ along with recent research into roles for paramedics,^28,29^ physicians associates,^30,31^ and other health professions.^32,33^ An early survey of patients indicated that a small number prefer access to the GP, which was demonstrated by the comment *‘like to see a doctor, call me old fashioned but you can’t make a silk purse from a sow's ear*’.^19^ However, there is overwhelming evidence from both this study and others that patients recognise the battle faced in general practice and are happy to accept the new roles as a resolution to the demand–supply challenge.^34^

The study demonstrated that, while some patients were clear about the role and its benefits, others were confused between the role of the pharmacist in the practice and in the community setting. This has parallels with findings from Karampatakis *et al*’s^20^ study into the views of community pharmacists, which also found role confusion. There are clear gaps between the development of new roles in the NHS and understanding of these by the public.^20,35^ The present study indicates further work is needed to raise awareness of the role and its benefits for patients as the implementation of the role develops longitudinally. This mirrors Karampatakis *et al,* ^20^ which pointed to limited publicity and awareness-raising about the role by NHSE or GP practices with the patients in their study.

The present study demonstrated that overall acceptability of the role by patients was high, in concert with findings from other studies.^15,19,20^

Patients felt pharmacist consultations were good quality and often better than those undertaken by GPs. This is consistent with evidence about medication reviews undertaken by pharmacists.^36–38^ Bradley *et al* ^15^ suggested that GPs rarely have time to perform an in-depth holistic medication review.

Good quality medication reviews can lead to better patient understanding, which leads to better adherence. This is fully consistent with literature around patient medication adherence.^36,39^ ^9^

Overall patients in the present study felt the pharmacists offered a more holistic service than GPs, which is consistent with studies about holistic consultations.^40^ Barnes *et al* ^9^ suggested that the increase in long-term conditions and ageing patients, and the need for holistic care identified a clear and necessary and unique role for pharmacists in medication management and adherence that works alongside others. There is evidence of this from the present study with the patient who was treated in a threesome by their GP, nurse, and pharmacist. Richards *et al* ^27^ suggested that the idea of GPs ‘delegating’ to another health professional needs to be replaced with the notion of integrated teams working together to address any implications of uneven power dynamics.

There was no evidence in the present study of pharmacists conducting physical examinations and this is an area that they would need to develop for clinical competency.^18^ Barnes *et al* ^9^ suggested that the ‘hands-on' examination role is more suited to the new physician’s associate role and pharmacists should concentrate on their expertise in communication around medication. However, it depends where the pharmacist is based and if they are part of a wide team.

Pharmacists were reported to deliver services through relationship building and consultation styles that build trust, consistent with the aims of the Royal College of General Practitioners towards continuity of care.^41^ This is a key priority for all professionals in general practice. Spending more time with patients and building relationships leads to increased adherence, which could in turn reduce hospital admissions.^9^

Patients recognised the value of the expertise that pharmacists can bring to the team through their ability to solve problems others could not. Pharmacists bring unique skills in medicines optimisation to the primary care team,^9^ and are a beneficial addition to the skill mix in general practice.^18^ This study has outlined interesting ways that a range of professional groups are working together for patient benefit in general practice, and the authors recommend further work to capture these exciting new ways of working. Many patients believed that the pharmacist role saved GP time, and this is evident in so far as the appointments would have to be conducted by an alternative prescriber. One study showed in 4 months, 5.4 (whole time equivalent) clinical pharmacists saved 628 GP appointments, and a further 647 hours of GP administration time.^12^ Williams *et al* estimate that having a clinical pharmacist in the primary healthcare team saves 80 GP-hours per month.^14^ However, this is a dichotomous advantage as there is clearly a cost for the pharmacist and their specialist expertise. It is important not to disregard this cost as it parallels with the value added to the team. Using GP-hours saved as a measure of effectiveness or justification creates an impression of pharmacists as *‘cheap doctors or expensive nurses’,* which does not acknowledge the wider benefits of the role, especially in relation to cost benefits of other outcomes, such as increased safety, reduced drug costs, and improved patient outcomes.^13^ As Hampson^16^ suggested: *‘Pharmacists are not there to act as cheap GPs; their role should complement that of GPs and other clinical staff.’*

Some patients already connected the idea of the pharmacist with the community pharmacy and were happy to seek services there. Some patients were willing to have consultations in the community setting, although there was evidence that patients would only access this service if there was access to their medical records. This is consistent with the debate in the literature that suggested that community pharmacists should have access to full patient records in order to facilitate better access to health services in the community^42^ and closer working relationships with general practice.

### Implications for research and practice

Pharmacists can add value to the general practice team and this is recognised by patients.

The study demonstrated a range of patient views and experiences of pharmacists in primary care. This work filled a gap in the literature by answering the research questions: what is the patient perspective of pharmacists in general practice in the UK?

Patients recognised the primary care crisis and the way that pharmacists can release capacity for GPs and use their expertise to fill a gap in the team. Patients reported a wide range of positive experiences and outcomes from interactions with pharmacists in primary care. Further work should be undertaken to raise awareness of the role and its benefits with patients, and to further evaluate the role and share the ongoing benefits as the rollout continues nationally across the UK.

Patients perceived that the pharmacist is a necessary and important part of the primary care skill mix. The study showed some confusion between the role and the community pharmacy and suggested that more work could be undertaken if there was access to patient medical records.

While many perceive the major benefit of the pharmacist is in the time ‘saved’ for GPs, this is a false dichotomy since pharmacists also have an associated cost, and the major benefit of the role is increasing access and adding unique expertise, enabling the patient better access to the right clinician at the right time.

Since the research was undertaken as the role was piloted and now it is deeply embedded, it is possible these perspectives may have changed; therefore, it will be important to follow-up the research with further studies on patient perspective to investigate more recent views.
